# Prognostic significance of ALK high expression in SCLC: a 9-year cohort analysis

**DOI:** 10.3389/fonc.2025.1530339

**Published:** 2025-03-25

**Authors:** Jinhe Xu, Wenting Zhang, Feilai Xie, Chenxi Wang, Feng Cheng, Ruiying Rao, Ying Chen, Lei Zhang, Wen Wen, Zhongquan Zhao, Jialing Yuan, Yuqin Zheng, Zongyang Yu

**Affiliations:** ^1^ Fuzong Clinical Medical College of Fujian Medical University, Fuzhou, China; ^2^ Pathology Department, Dongfang Hospital of Xiamen University, Fuzhou General Hospital of Fujian Medical University, The 900th Hospital of the Joint Logistic Support Force, PLA, Fuzhou, China; ^3^ Department of Respiratory and Critical Care Medicine, Fuzong Teaching Hospital, Fujian University of Traditional Chinese Medicine (900 Hospital), Fuzhou, China; ^4^ Department of Pulmonary and Critical Care Medicine, Fuzhou General Hospital of Fujian Medical University, Dongfang Hospital of Xiamen University, The 900th Hospital of the Joint Logistic Support Force, PLA, Fuzhou, China

**Keywords:** small cell lung cancer, ALK, immunohistochemistry, 5D3F, prognosis

## Abstract

**Purpose:**

The aim of this study was to investigate the prognostic value of the abnormal expression of anaplastic lymphoma kinase (ALK) protein in patients with small cell lung cancer (SCLC) based on 9-year data from our center.

**Methods:**

A retrospective cohort study was conducted to assess the clinical outcomes of patients with ALK-positive SCLC diagnosed in our hospital over the past 9 years. We used public databases to analyze the expression of ALK in pan-cancer and its prognostic value and analyzed the correlation between ALK and SCLC prognosis-related genes.

**Results:**

A total of 685 patients diagnosed with SCLC underwent ALK testing, and 59 patients were identified to have abnormal expression of the ALK protein, with 10 cases showing strong expression, 14 cases displaying moderate expression, and 35 cases exhibiting weak expression. The median age of the ALK-positive cohort was 64 years (range: 58–70 years), 91.5% (54/59) were male, 61.0% (36/59) were smokers, and the median overall survival (mOS) was 7.0 months (95% CI: 4.5–9.5 months). Within this cohort, the mOS for the ALK (+) subgroup was 4.0 months (95% CI: 2.9–5.1 months), the mOS for the ALK (++) subgroup was 10.0 months (95% CI: 4.9–15.1 months), and the mOS for the ALK (+++) subgroup was 12.0 months (95% CI: 7.4–16.6 months). Kaplan–Meier revealed that the mOS of the ALK_Low_ group was significantly worse than that of the ALK_High_ group [mOS: 4.0 months (95% CI: 2.9–5.1 months) versus 11.0 months (95% CI: 8.3–13.7 months), *p* = 0.009]. Following covariate adjustment using a Cox regression model, it was indicated that the level of abnormal expression of the ALK protein was an independent prognostic factor for patients with SCLC (HR: 0.486, 95% CI: 0.271–0.871, *p* = 0.015).

**Conclusion:**

The prognosis for patients with SCLC with strong abnormal expression of the ALK protein was significantly better than those with weak expression.

## 1 Introduction

Lung cancer ranks among the malignancies with the highest incidence and mortality rates both nationally and globally (1), of which small cell lung cancer (SCLC) accounts for 13%–15%, which has a poor prognosis, is highly invasive, and is related to smoking status (2, 3). SCLC is primarily classified into limited-stage and extensive-stage disease (4). Early prognostic assessment of SCLC could facilitate the development of an appropriate treatment strategy, thereby enabling proactive intervention to enhance quality of life and extend survival. Over the past three decades, there has been significant progress in the development of targeted therapies aimed at specific driver genes that have markedly extended the survival of patients with advanced non-small cell lung cancer (NSCLC) who test positive for these genetic alterations (5). Anaplastic lymphoma kinase (ALK) is present in 5%–6% of NSCLC cases, with common aberrations including ALK fusions, mutations, and amplifications (6). The ALK fusion gene is colloquially referred to as a “masonry mutation” due to its low prevalence in NSCLC; however, patients with this mutation can derive substantial clinical benefits from ALK-tyrosine kinase inhibitors (ALK-TKIs), potentially achieving prolonged survival (6). Because of the rarity of targeted gene mutations in SCLC and its insensitivity to targeted drugs, routine testing for gene mutations in SCLC is currently not recommended clinically (7). Nonetheless, some studies suggested that aberrant ALK protein expression may be associated with the prognosis of some tumors. In a study that examined the prognosis of gastric cancer, it was observed that an increase in the intensity of ALK protein expression correlated with a higher proportion of tumor signet ring cells and a poorer prognosis (8). ALK protein expression has been shown to predict micrometastasis and unfavorable outcomes in patients with hepatocellular carcinoma (9). Conversely, elevated ALK protein expression is associated with prolonged survival in patients with Merkel cell carcinoma (MCC) and anaplastic large cell lymphoma (ALCL) (10, 11). ALK immunohistochemistry (IHC) serves as a standard clinical marker for SCLC. Previous research has identified that the protein expression rate of ALK in SCLC can reach 11% (12). However, there is no research that investigated the prognostic significance of aberrant ALK protein expression in SCLC. This study undertook a retrospective analysis of 685 patients with SCLC and their tissue samples collected over the past 9 years. The objectives are to examine the clinical characteristics associated with positive abnormal ALK protein expression and to evaluate the prognostic implications of ALK protein expression levels in SCLC.

## 2 Materials and methods

### 2.1 Patients

**Figure 1:**
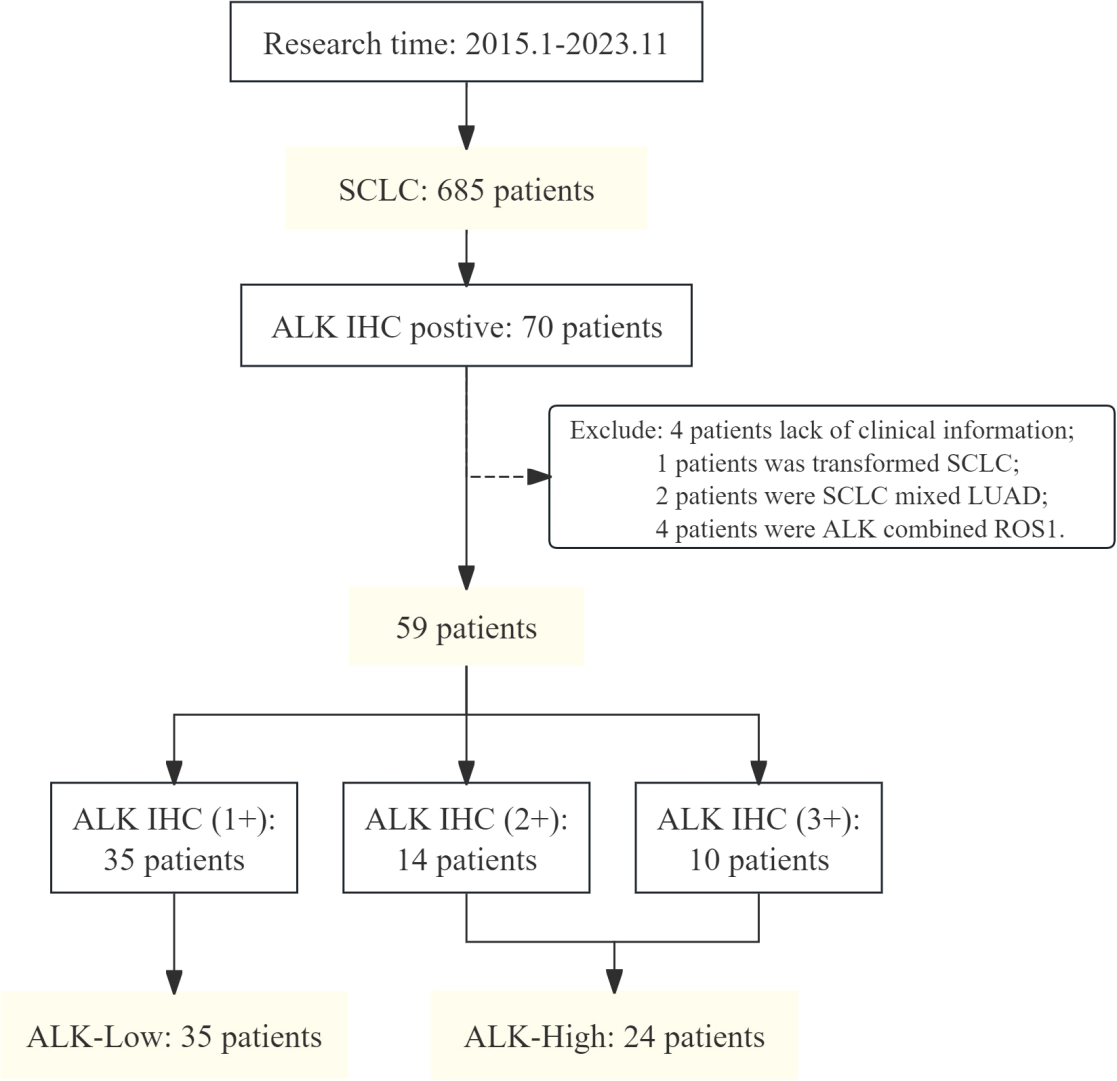
Flowchart of patient enrollment. SCLC, small cell lung cancer; ALK, anaplastic lymphoma kinase; IHC, immunohistochemistry; LUAD, lung adenocarcinoma; ROS1, ROS proto-oncogene 1, receptor tyrosine kinase.

A total of 685 eligible patients with *de novo* SCLC who were subjected to ALK IHC for analysis at the 900th Hospital of the Joint Logistic Support Force of China (Fujian, China) between January 2015 and November 2023 were enrolled in this study. All patients had a *de novo* diagnosis of SCLC excluding the histological transformation types. Among them, 35 patients with SCLC with weak positive ALK IHC (1+) were classified as ALK_Low_, while 24 patients with strong positive ALK IHC (2+, 3+) were categorized as ALK_High_ (**Figure 1**). In this series, all samples were detected by ALK IHC at the initial biopsy. Histologic diagnosis of SCLC was based on the standard criteria defined by the WHO classification 2015 version (13). All procedures performed in this study involving human participants were in accordance with the Declaration of Helsinki (as revised in 2013). This study was approved by the Ethics Committee of the 900th Hospital of the Joint Logistic Support Force of China.

### 2.2 Data collection and outcome assessment

The following information was retrospectively collected from the medical records of the patients: patient demographics such as sex, age, smoking history, disease stage, anatomy type, histological type, systemic therapies, tumor imaging, and tumor response to therapies. Tumor response was assessed in accordance with the Response Evaluation Criteria in Solid Tumors (RECIST version 1.1) (14). Overall survival (OS) was defined as the time from initial therapy to death, and the last follow-up was on 15 September 2024.

### 2.3 Immunohistochemistry staining

**Figure 2:**
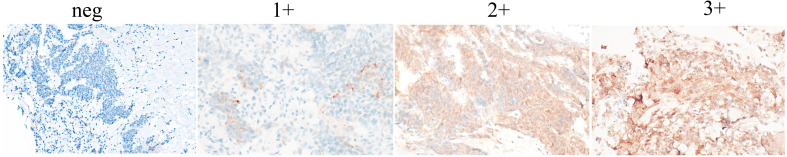
ALK immunohistochemistry staining pattern. Examples of negative and positive (1+, 2+, and 3+) staining patterns of ALK immunohistochemistry are shown (hematoxylin and eosin stain, ×400). neg, negative.

IHC stains were conducted on an automated immunostainer machine. IHC staining was performed on 4-μm sections obtained from formalin-fixed and paraffin-embedded tissue blocks and then mounted on charged slides. After deparaffinization and rehydration, antigen retrieval was performed with Cell Condition Solution-1 (CC1) for 64 min at 95°C. ALK IHC assay was performed using Ventana anti-ALK rabbit monoclonal primary antibody (clone D5F3, Cell Signaling Technology). The expression level of ALK was expressed using IHC scoring, and the expression of all samples was scored by two experienced pathologists (**Table 1**). When using the four-tiered scoring system, cases showing no staining were considered as negative, cases with 2+/3+ staining were considered as positive/moderate and strong, and cases with 1+ intensity expression were considered as weak. The representative images of immunohistochemical markers are shown in **Figure 2**.

**Table 1:** ALK IHC staining: scoring system based on spread and intensity.

Spread (P)	Strength (I)		
Cell percentage	Score	Level/Intensity	Fraction
0	0	None	0
1% ≦ 24%	1+	Weak	1+
25% ≦ 49%	2+	Mitigated	2+
50%–100%	3+	Strong	3+

The threshold for high ALK expression was defined as at least 25% of tumor cells staining with an intensity of 2+ or 3+ (P score of 2+ or 3+). All other positive expression patterns or intensities were defined as low ALK expression.

### 2.4 Statistical analysis

Continuous variables were analyzed as the median and interquartile range (IQR) or mean and standard deviation (mean ± SD), and differences were compared using *t*-test. Categorical data were calculated as the frequency (percentage) and composition ratio (%), and non-parametric methods such as the χ^2^ test were used to compare differences. Survival analysis was performed using the Kaplan–Meier method, and the log-rank test was used to determine the significance of differences between two subgroups of patients. Single-factor and multi-factor Cox proportional hazards regression models were used to analyze the relationship between survival time and survival status and factors. Among them, dead and alive are used as dependent variables, and other variables are used as independent variables. IBM SPSS Statistics (Armonk, NY), version 26 was used in statistical analyses. A *p*-value < 0.05 was considered significant, while all tests were two-sided.

## 3 Results

### 3.1 Clinical features

A total of 685 eligible patients diagnosed with SCLC at our institution were included in our study; 70 cases showed positive ALK protein expression in IHC test results, with an incidence rate of 10.22%. The following cases were excluded from the study: four cases with incomplete information, two cases of mixed tissue type, four cases with other mutations, and one case of transformed SCLC. Finally, 59 patients were included in the follow-up study. The median age was 64 years (range: 58–70 years), 54 patients (91.5%) were men, and 36 patients (61.0%) were smokers. Among the cohort, 35 patients exhibited weak ALK protein expression (+), 14 patients demonstrated moderate expression (++), and 10 patients showed strong expression (+++). Comparative analysis of the different ALK protein expression groups revealed no statistically significant differences in age, gender, smoking history, TNM stage, primary lesion site, or Ki-67 expression. Regarding first-line treatment, aside from those detailed in **Table 2**, two additional patients received only chest radiotherapy (ALK 1+), and one patient underwent alectinib targeted therapy (ALK 2+). No statistically significant differences were observed in the first-line treatment outcomes in different ALK protein groups.

**Table 2:** Clinical characteristics.

Characteristic	Total	ALK protein expression			*p*-value
	*N* = 59	1+ (*N* = 35)^1^	2+ (*N* = 14)^1^	3+ (*N* = 10)^1^	
Age					0.854^2^
Mean ± SD Median (IQR) Range	64 ± 8 64 (58, 70) 43, 81	63 ± 10 64 (56, 71) 43, 79	65 ± 5 65 (60, 70) 57, 72	65 ± 8 65 (60, 68) 55, 81	
Gender					0.443^3^
Female Male	5 (8.5%) 54 (91.5%)	4 (11.4%) 31 (88.6%)	0 (0.0%) 14 (100.0%)	(10.0%) 9 (90.0%)	
Smoking					0.810^3^
No Yes	23 (39.0%) 36 (61.0%)	15 (42.9%) 20 (57.1%)	5 (35.7%) 9 (64.3%)	3 (30.0%) 7 (70.0%)	
TNM stage					0.249^3^
III IV	16 (27.1%) 43 (72.9%)	7 (20.0%) 28 (80.0%)	6 (42.7%) 8 (57.1%)	3 (30.0%) 7 (70.0%)	
Primary tumor site					0.643^3^
Right lobe Right hilum Left lobe Left hilum	20 (33.9%) 11 (18.6%) 16 (27.1%) 12 (20.3%)	13 (37.1%) 5 (14.3%) 10 (28.6%) 7 (20.0%)	4 (28.6%) 2 (14.3%) 5 (35.7%) 3 (21.4%)	3 (30.0%) 4 (40.0%) 1 (10.0%) 2 (20.0%)	
First-line treatment					0.219^3^
Chem Chem + Radi Chem + Imm Chem + Imm + Radi None	13 (22.0%) 12 (20.3%) 7 (11.9%) 5 (8.5%) 19 (32.2%)	7 (20.0%) 5 (14.3%) 4 (11.4%) 2 (5.7%) 15 (42.9%)	4 (28.6%) 4 (28.6%) 2 (14.3%) 1 (7.1%) 2 (14.3%)	2 (20.0%) 3 (30.0%) 1 (10.0%) 2 (20.0%) 2 (20.0%)	

^1^ *n* (%), ^2^Kruskal–Wallis rank sum test, ^3^Fisher’s exact test; Chem, chemotherapy; Radi, radiotherapy; Imm, immunotherapy; TNM, tumor node metastasis classification; None, no treatment.

### 3.2 Immunohistochemistry analysis

The heterogeneity of SCLCs is substantial, and they are not simply a result of combining multiple subtypes or the presence of different mutant molecules. Therefore, we examined whether there is a discernible histological inclination between different ALK protein expression levels in 59 patients with ALK IHC-positive SCLC using IHC. Results of the IHC analysis are shown in **Table 3**. The results of IHC were interpreted using semiquantitative criteria; Ki-67 results determined the positive rate of tumor cell nuclei. The expression of CD56 was statistically significant in different ALK IHC expression groups (*p* < 0.05), and the difference was mainly in the ALK IHC (2+) group. However, the expression of CK7, CgA, Syn, TTF-1, and Ki-67, and the proportion of patients with marker 0, 1, 2, 3, and 4 did not differ significantly among the groups, which means the IHC characteristics of different ALK IHC expression groups were similar in general.

**Table 3:** IHC characteristics of different ALK IHC expression level groups in patients with SCLC.

Characteristic	ALK IHC expression			*p*-value
	1, *N* = 35^1^	2, *N* = 14^1^	3, *N* = 10^1^	
Ki-67	81 ± 12	82 ± 9	86 ± 7	0.452^2^
CK7				>0.999^3^
0	14 (45.2%)	5 (45.5%)	5 (50.0%)	
1+	5 (16.1%)	2 (18.2%)	2 (20.0%)	
2+	7 (22.6%)	2 (18.2%)	2 (20.0%)	
3+	4 (12.9%)	2 (18.2%)	1 (10.0%)	
4+	1 (3.2%)	0 (0.0%)	0 (0.0%)	
CgA				0.705^3^
0	15 (68.2%)	6 (75.0%)	3 (75.0%)	
1+	3 (13.6%)	2 (25.0%)	1 (25.0%)	
2+	4 (18.2%)	0 (0.0%)	0 (0.0%)	
Syn				0.790^3^
1+	2 (5.7%)	1 (7.1%)	0 (0.0%)	
2+	8 (22.9%)	5 (35.7%)	2 (20.0%)	
3+	17 (48.6%)	7 (50.0%)	5 (50.0%)	
4+	8 (22.9%)	1 (7.1%)	3 (30.0%)	
TTF-1				0.423^3^
0	1 (2.9%)	0 (0.0%)	1 (10.0%)	
1+	1 (2.9%)	0 (0.0%)	1 (10.0%)	
2+	2 (5.9%)	1 (7.7%)	0 (0.0%)	
3+	12 (35.3%)	6 (46.2%)	6 (60.0%)	
4+	18 (52.9%)	6 (46.2%)	2 (20.0%)	
CD56				0.003^3^
0	0 (0.0%)	3 (21.4%)	0 (0.0%)	
1+	0 (0.0%)	1 (7.1%)	0 (0.0%)	
2+	0 (0.0%)	1 (7.1%)	1 (11.1%)	
3+	15 (50.0%)	6 (42.9%)	1 (11.1%)	
4+	15 (50.0%)	3 (21.4%)	7 (77.8%)	

Semiquantitative criteria: no staining or sporadic (<1%) positive was negative, 1%–24% was +, 25%–49% was ++, 50%–75% was +++, and >75% was ++++; infiltrating inflammatory cells/lymphocytes covering the lesion area <25% was +, 25%–49% was ++, 50%–75% was +++, and >75% was ++++. Ki-67 counted the positive rate of tumor cell nuclei. Ki-67, proliferation cell nuclear antigen; CK7, cytokeratin7; CgA, chromogranin A; Syn, synaptophysin; TTF-1, thyroid transcription factor-1; CD56, neural cell adhesion molecule 1; ^1^Mean ± SD; *n* (%); ^2^One-way ANOVA; ^3^Fisher’s exact test.

### 3.3 Survival analysis

**Figure 3:**
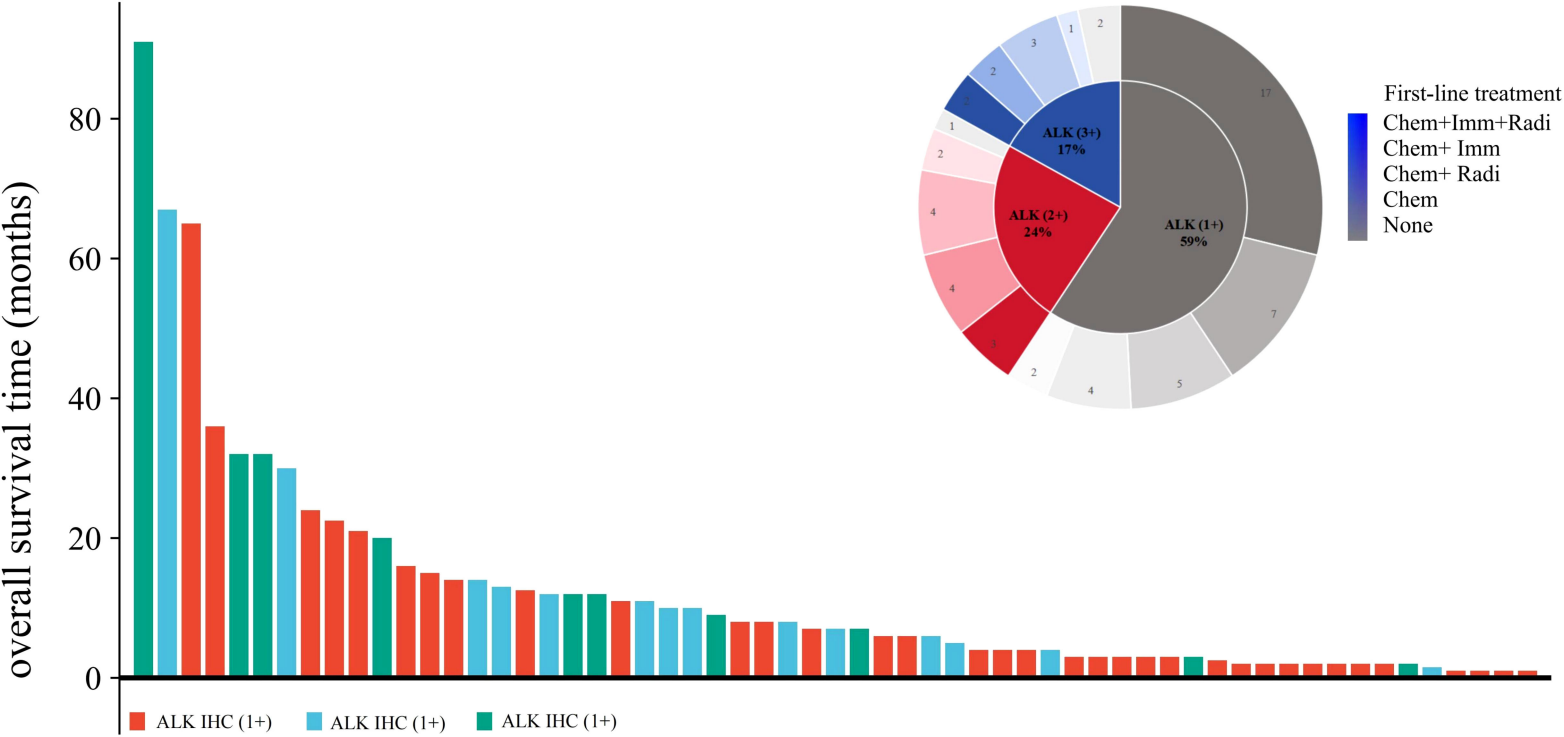
Patient survival swim lane diagram of this study cohort. Chem, chemotherapy; Radi, radiotherapy; Imm, immunotherapy; None, no treatment.

**Figure 4:**
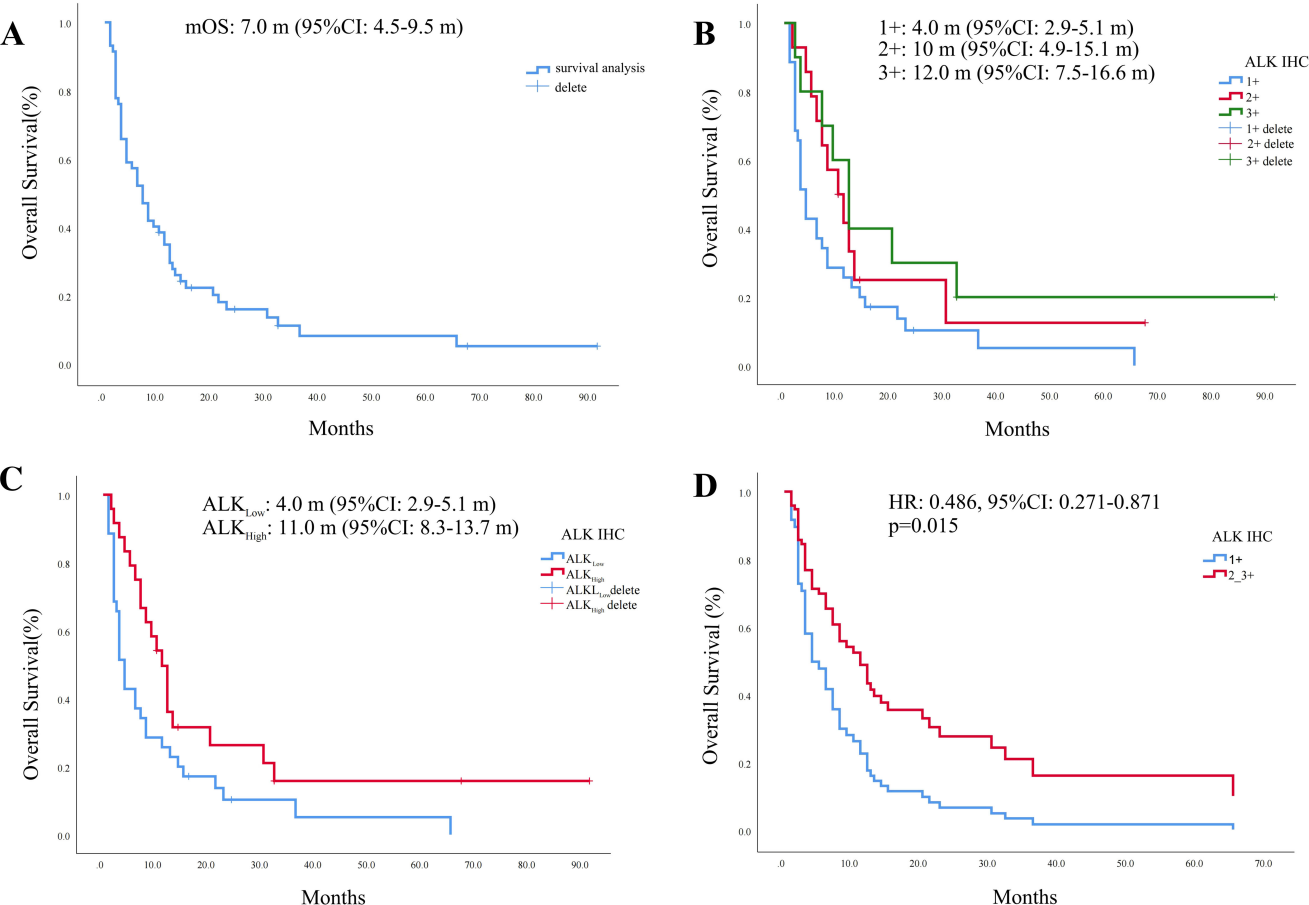
Kaplan–Meier survival curve. **(A)** Kaplan–Meier curve of the overall population. **(B)** Kaplan–Meier curve of different ALK protein expression groups. **(C)** Survival analysis of ALK_Low_ and ALK_High_ groups. **(D)** ALK protein Cox survival curve after adjusting for clinical parameter covariates.

In our study cohort, 88% (52/59) of the patients had OS. Here, we used a swim lane diagram to show the specific survival situation of the patients in the study cohort (**Figure 3**). Kaplan–Meier analysis showed that the overall population median OS (mOS) was 7.0 months [95% confidence interval (CI): 4.5–9.5 months] (**Figure 4A**). Among them, the mOS of the ALK (+) group was 4.0 months (95% CI: 2.9–5.1 months), the mOS of the ALK (++) group was 10.0 months (95% CI: 4.9–15.1 months), and the mOS of the ALK (+++) group was 12.0 months (95% CI: 7.5–16.6 months). Kaplan–Meier analysis showed that there were significant differences in prognosis between groups with different levels of ALK protein expression (*p* = 0.031) (**Figure 4B**).

Since the number of cases with moderate to strong and weak expression of ALK protein in our study cohort was relatively small, the cohort population was regrouped into two groups—the weak expression group (ALK IHC 1+, ALK_Low_) and the moderate to strong expression group (ALK IHC 2+_3+, ALK_High_)—for comparison. General clinical characteristics showed that there were still no statistical differences between the two groups in terms of age, gender, smoking history, TNM stage, primary tumor site, and Ki-67 expression (**Table 4**). Survival analysis showed that the mOS of the ALK_Low_ group was significantly worse than that of ALK_High_ [mOS: 4.0 months (95% CI: 2.9–5.1 months) vs. 11.0 months (95% CI: 8.3–13.7 months), *p* = 0.009] (**Figure 4C**). After covariate adjustment analysis of different clinical parameters used the Cox regression model, the results showed that ALK protein expression level was an independent factor that affected the prognosis of patients with SCLC (HR: 0.486, 95% CI: 0.271–0.871, *p* = 0.015) (**Figure 4D**).

**Table 4:** Clinical characteristics of ALK_Low_ and ALK_High_ groups.

Characteristic	ALK protein expression		*p*-value
	ALK_Low_ (*N* = 35)^1^	ALK_High_ (*N* = 24)^1^	
Age			
Mean ± SD Median (IQR) Range	63 ± 10 64 (56, 71) 43, 79	65 ± 6 65 (60, 69) 55, 81	0.694^2^
Gender			
Female Male	4 (11.4%) 31 (88.6%)	1 (4.2%) 23 (95.8%)	0.639^3^
Smoking			
No Yes	15 (42.9%) 20 (57.1%)	8 (33.3%) 16 (66.7%)	0.639^3^
TNM stage			
III IV	7 (20.0%) 28 (80.0%)	9 (37.5%) 15 (62.5%)	0.137^4^
Primary tumor site			
Right lobe Right hilum Left lobe Left hilum	13 (37.1%) 5 (14.3%) 10 (28.6%) 7 (20.0)	7 (29.2%) 6 (25.5%) 6 (25.5%) 5 (20.8%)	0.760^3^
First-line treatment			
Chem Chem + Radi Chem + Imm Chem + Imm + Radi None	13 (22.0%) 12 (20.3%) 7 (11.9%) 5 (8.5%) 19 (32.2%)	6 (25.0%) 7 ((29.2%) 3 (12.5%) 3 (12.5%) 4 (16.7%)	0.219^3^
Ki-67			
Mean ± SD Median (IQR) Range	81 ± 12 85 (80, 90) 40, 95	84 ± 8 90 (80, 90) 65, 90	0.536^2^

^1^ *n* (%), ^2^Wilcoxon rank sum test, ^3^Fisher’s exact test, ^4^Pearson’s chi-squared test; Chem, chemotherapy; Radi, radiotherapy; Imm, immunotherapy; None, no treatment.

## 4 Discussion

ALK fusion is a significant molecular event observed in approximately 5% of NSCLC cases, particularly in adenocarcinoma (15). Identifying patients with ALK fusion is crucial, as they derive significant benefits from ALK-TKIs, which have demonstrated remarkable efficacy in this patient group (16–18). Different from NSCLC, owing to rapid growth and early metastatic spread, SCLC does not benefit from low-dose computed tomography (CT) screening (19, 20). OS for the extended disease is extremely poor (<10% at 5 years) (21). The standard approach has involved platinum-based chemotherapy, resulting in rapid and profound responses but rarely achieving long-term durability (21). Recent translational research has begun to challenge this paradigm, fueling increasing interest in the molecular subtypes of SCLC and their potential implications for therapeutic strategies (22, 23).

The ALK (D5F3) IHC assay, while not a molecular test, demonstrates high sensitivity and specificity in detecting ALK fusions, making it a valuable diagnostic tool in clinical settings as companion diagnostics (24–27). Among the companion diagnostics for detecting ALK fusion, fluorescence *in situ* hybridization (FISH) was the first method to be clinically validated (28). Another molecular method for detecting ALK fusion is next-generation sequencing (NGS), which allows the parallel examination of millions or billions of deoxyribonucleic acid (DNA) strands. NGS has the advantage of simultaneously detecting multiple driver gene mutations across a wide range. Previous studies have demonstrated that NGS exhibits high sensitivity and specificity for ALK fusion detection (29). However, the turnaround time for NGS is over 2 weeks, which is longer than that of other tests, and it is more expensive, presenting certain limitations. The Ventana ALK (D5F3)-approved ALK IHC assay provides high analytical sensitivity and specificity, high concordance with FISH, and very high interobserver reproducibility (30, 31). The D5F3 clone detects the carboxyl terminus of the ALK protein, and numerous studies have demonstrated its excellent performance (24). In a series of 142 consecutive cases of treatment-naïve SCLC, 11% (*n* = 16) showed expression of ALK in IHC (12). The expression rate of ALK IHC in our cohort was 10.22%, which is similar to that in this study. Compared with the diffuse high-intensity expression generally shown in NSCLC, ALK expression was focal and of low intensity: 59.3% were weakly expressed (+), 23.7% were moderately expressed (++), and 16.9% were strongly expressed (+++).

ALK gene alterations are gaining more attention as pan-cancer markers in both solid and hematological malignancies (32). ALK genomic alterations are found in ~3.3% of patients with cancer, though ALK fusions/rearrangements are less common (33). In large-scale analyses of genomes, ALK fusions/rearrangements are detected in ~0.5%–0.8% of cancers (33, 34). In order to explore the expression of ALK in other tumors and its impact on prognosis, we downloaded the unified normalized pan-cancer dataset (TCGA, TARGET, and GTEx) from the UCSC (https://xenabrowser.net/) database, from which we extracted ENSG00000171094 (ALK) gene expression data in various samples. It was found that the expression rate of ALK in cancer patients was 0.2%–10.3%, significant upregulation of ALK expression was observed in 16 tumor types (GBM, LGG, UCEC, KIPAN, KIRC, LIHC, WT, SKCM, THCA, OV, PAAD, UCS, LAML, PCPG, KICH, and CHOL), and significant downregulation was observed in 11 tumor types (BRCA, ESCA, STES, COAD, COADREAD, PRAD, STAD, BLCA, READ, TGCT, and ACC) (**Figure 1**). In addition, we analyzed the prognostic relationship between ALK expression and each tumor by an established Cox proportional hazards regression model, and we used the log-rank test for statistical testing to obtain prognostic significance. The results showed that ALK expression was negatively correlated with the prognosis of LAML [*p* < 0.001, HR = 1.10 (1.05, 1.16)], STAD [*p* = 9.8e-3, HR = 1.10 (1.02, 1.18)], NB [*p* = 0.04, HR = 1.17 (1.01, 1.36)], and UVM [*p* = 8.4e-3, HR = 1.37 (1.08, 1.74)], and positively correlated with the prognosis of CESC [*p* = 0.01, HR = 0.90 (0.83, 0.98)], KIRP [*p* = 0.02, HR = 0.89 (0.81, 0.99)], SKCM [*p* = 8.2e-3, HR = 0.91 (0.85,0.98)], and DLBC [*p* = 0.04, HR = 0.72 (0.53, 0.97)] (**Figure 2**).

A review of previously published studies found that ALK expression is indeed associated with improved prognosis in some tumors. Previous studies have found that inflammatory myelofibrotic tumors (IMTs) are associated with ALK gene rearrangement in approximately 50% of cases (35). ALK-overexpressing IMTs may have a better prognosis than ALK non-expressing tumors (36, 37). In 2015, Chou et al. detected ALK translocation in 2.2% of patients with papillary thyroid cancer and believed that it was associated with a good prognosis of thyroid cancer (38). Actually, ALK gene rearrangements/fusions are more commonly found in hematological malignancies such as ALCL (32). Earlier studies have shown that ALK-expressing ALCL has a much better 5-year OS rate (70%–80% vs. 15%–45%) and 5-year failure-free survival (FFS: 60% vs. 36%; *p* = 0.015) than non-ALK-expressing ALCL (11, 39) and found that in high-grade serous ovarian cancer (HGSOC), HGSOC harboring activating ALK mutations might be associated with a better survival, while ALK overexpression and ALK amplification do not impact the prognosis (40). Similarly, in MCC, the presence of ALK and phosphorylated ALK (p-ALK) was determined by immunohistochemistry, with almost half of the analyzed MCC tumors displaying ALK phosphorylation (47.8%). Survival analysis showed that p-ALK in MCC was associated with longer survival, with intermediate/high ALK and p-ALK tumor expression having better survival (10). In addition, a study that explored prognosis-related clinical and molecular factors in malignant pleural mesothelioma found that the median survival time (MST) of EML4-ALK-positive patients was longer than that of negative ones (19.6 months vs. 9.57 months), although no statistical significance was seen (*p* = 0.159), but the authors considered it to be related to the small sample size (41).

In the past 9 years, we found that 70 patients were identified as having abnormal expression of the ALK protein in SCLC among the 685 patients diagnosed with SCLC. In our study, we used the results of ALK IHC to reclassify SCLC and evaluated the correlation between the nonspecific expression of the ALK protein and the prognosis of patients with SCLC. We divided the patients into an ALK weak expression group (ALK_Low_) and an ALK moderate to strong expression group (ALK_High_) according to the ALK protein expression level on IHC. Survival analysis showed that the prognosis of the ALK_Low_ group was significantly worse than that of the ALK_High_ group [mOS: 4.0 months (95% CI: 2.9–5.1 months) vs. 11.0 months (95% CI: 8.3–13.7 months), *p* = 0.009], which suggested that high expression of the ALK protein is a better prognostic marker for SCLC. To our knowledge, this was the first clinical study on the prognostic significance and value of ALK protein expression directly evaluated by IHC in SCLC. Indeed, differential gene expression in the different molecular subtypes and during the disease course might influence sensitivity and resistance to several therapeutic agents (42). In recent years, our understanding of molecular profiling for SCLC has been steadily expanding, revealing potential genetic alterations that can be identified via various molecular biology techniques and panels despite the absence of approved targeted treatments for the disease (43). Previous studies have found that epidermal growth factor receptor (EGFR)-mutated patients with SCLC had longer OS than EGFR wild type even though they were not treated with EGFR-TKI, suggesting a potential favorable prognostic role of EGFR mutations in SCLC (7). Moreover, the expression of KRAS G12C, BRAF, NF1, NEUROD1, ASCL1, SOX2, CYP1B1, SLIT2, CDK6, GCLC, and NFYA genes is also associated with improved prognosis of SCLC (7, 42, 44–49). Our study suggests that identifying ALK protein expression in SCLC has significant prognostic value. Through correlation network analysis, we found that the expression of ALK was positively correlated with KRAS, BRAF, NF1, ASCL1, SOX2, CYP1B1, SLIT2, CDK6, and GCLC (**Figure 3**), suggesting that the mechanism by which high expression of ALK improves the prognosis of SCLC may be related to them.

The limited experiences of targeted therapies for the treatment of SCLC regard almost exclusively the use of EGFR and ALK inhibitors in the presence of specific molecular alterations of the corresponding genes. EGFR mutations and ALK rearrangements have been described in cases of histological transformation as a mechanism of resistance to TKIs, but *de novo* alterations of these genes in SCLC are unusual (50–53). As mentioned in a previous study, a recent large-scale NGS analysis was performed on 3,600 real-world SCLC cases. Besides EGFR mutations, ALK (*n* = 5), RET (*n* = 5), ROS1 (*n* = 3), and NTRK1 (*n* = 1) oncogenic molecular alterations were also detected (54). The characteristics in this study cohort showed a similar distribution based on sex; most patients had a smoking history (96.5%) and exhibited advanced-stage disease at the time of initial diagnosis (82.6%). mOS for the cohort was 8.0 months (95% CI: 7.3–9.0 months). However, in our study cohort, most patients were men (91.53%), and 61.02% (36/59) were smokers. The mOS of the cohort was 7.0 months (95% CI: 4.5–9.5 months). Subgroup analysis of our cohort suggested that high ALK IHC expression was associated with increased OS in patients with SCLC (HR: 0.486, 95% CI: 0.271–0.871, *p* = 0.015).

Furthermore, as regards the possibility of using ALK-TKI as targeted therapy in selected cases of SCLC, a clinical case reported rapid partial response (PR) to alectinib combined with irinotecan as second-line treatment for a 26-year-old patient with SCLC, and the progression-free survival (PFS) reached 6 months (55). A 38-year-old male SCLC patient with EML4-ALK fusion confirmed by IHC, FISH, and direct sequencing received crizotinib as first-line treatment and achieved PR, but the PFS and OS were not reported in the article (56). In one case involving a patient with SCLC, NGS detected a novel pleckstrin homology and RUN domain containing M2 (PLEKHM2)-ALK fusion; the patient experienced long-lasting clinical benefit after treatment with a combination of standard chemotherapy and crizotinib, achieving an OS of more than 27 months (57). However, in our cohort, one patient with ALK (++) experienced rapid progression after first-line targeted therapy with alectinib, with an OS of only 5 months. In a study that retested SCLC with ALK IHC expression using FISH and polymerase chain reaction (PCR), no activating alterations (rearrangements, point mutations, or amplification) were detected, and in the cases where copy number gains were shown (4/12), they were mild, accounting for three to five copy increases (12). The absence of activating mutations behind the expression of the protein suggests that only a normal form of ALK is expressed. Thus, ALK expression should not be considered as a surrogate for the presence of a molecular target in SCLC (58). Based on these experiences, we propose that patients with ALK-positive SCLC may benefit from chemotherapy combined with ALK-TKI rather than ALK-TKI targeted therapy alone.

In conclusion, our study innovatively evaluated the prognostic value of ALK abnormal protein expression levels in patients with SCLC when ALK IHC was positive. Interestingly, elevated expression levels of the ALK protein were significantly correlated with favorable prognosis in patients with SCLC. It is important to acknowledge that this investigation was conducted as a single-center retrospective study, which may introduce potential biases and limit the representativeness of the sample. To address these limitations and validate the findings, our center is spearheading a multicenter retrospective study aimed at further assessing the reliability of these conclusions. Additionally, our analysis revealed that the intensity ALK protein expression did not exhibit any significant association with various clinical and histopathological characteristics. Future studies are needed to elucidate the association between ALK expression and ALK gene status and to investigate disease progression, especially the oncogenesis of ALK-positive SCLC.

## Data Availability

The original contributions presented in the study are included in the article/**Supplementary Material**. Further inquiries can be directed to the corresponding author.
