# Supplementary material for: Prognostic significance of ALK high expression in SCLC: a 9-year cohort analysis
Source: Front Oncol. 2025 Mar 25;15:1530339. doi: 10.3389/fonc.2025.1530339 (PMC11975910; doi:10.3389/fonc.2025.1530339)
Supplement: Supplementary file 1 [file DataSheet1.pdf]

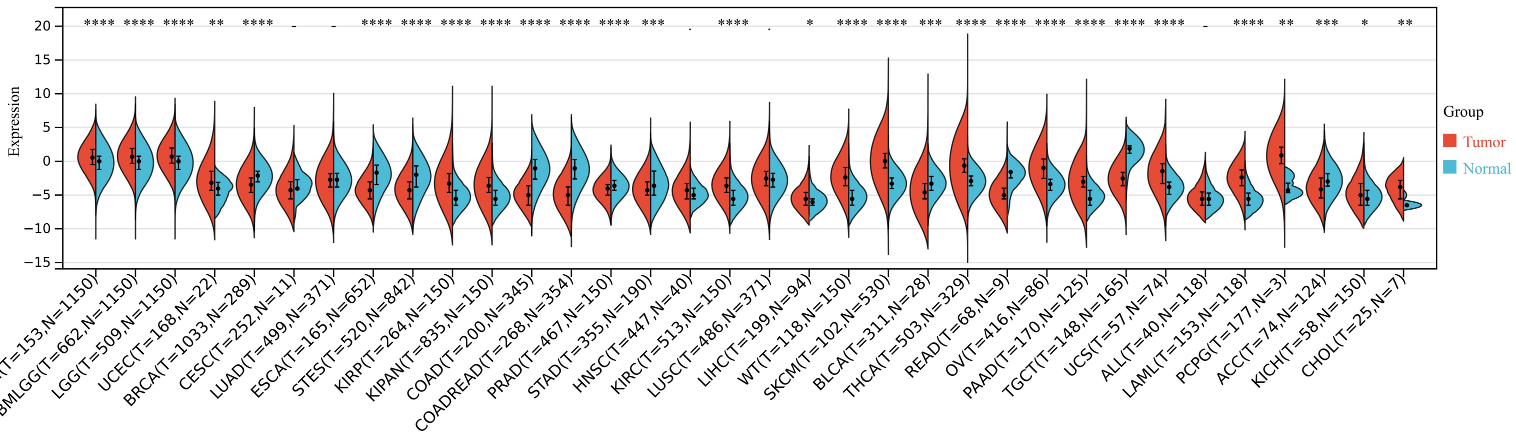

Attached figure 1: Analysis of differential expression of ALK in pan-cancer.
Difference significance analysis was performed using unpaired Wilcoxon Rank Sum and Signed Rank Tests; *, p<0.05; **, p<0.01; ***, p<0.001; ****, p<0.0001.

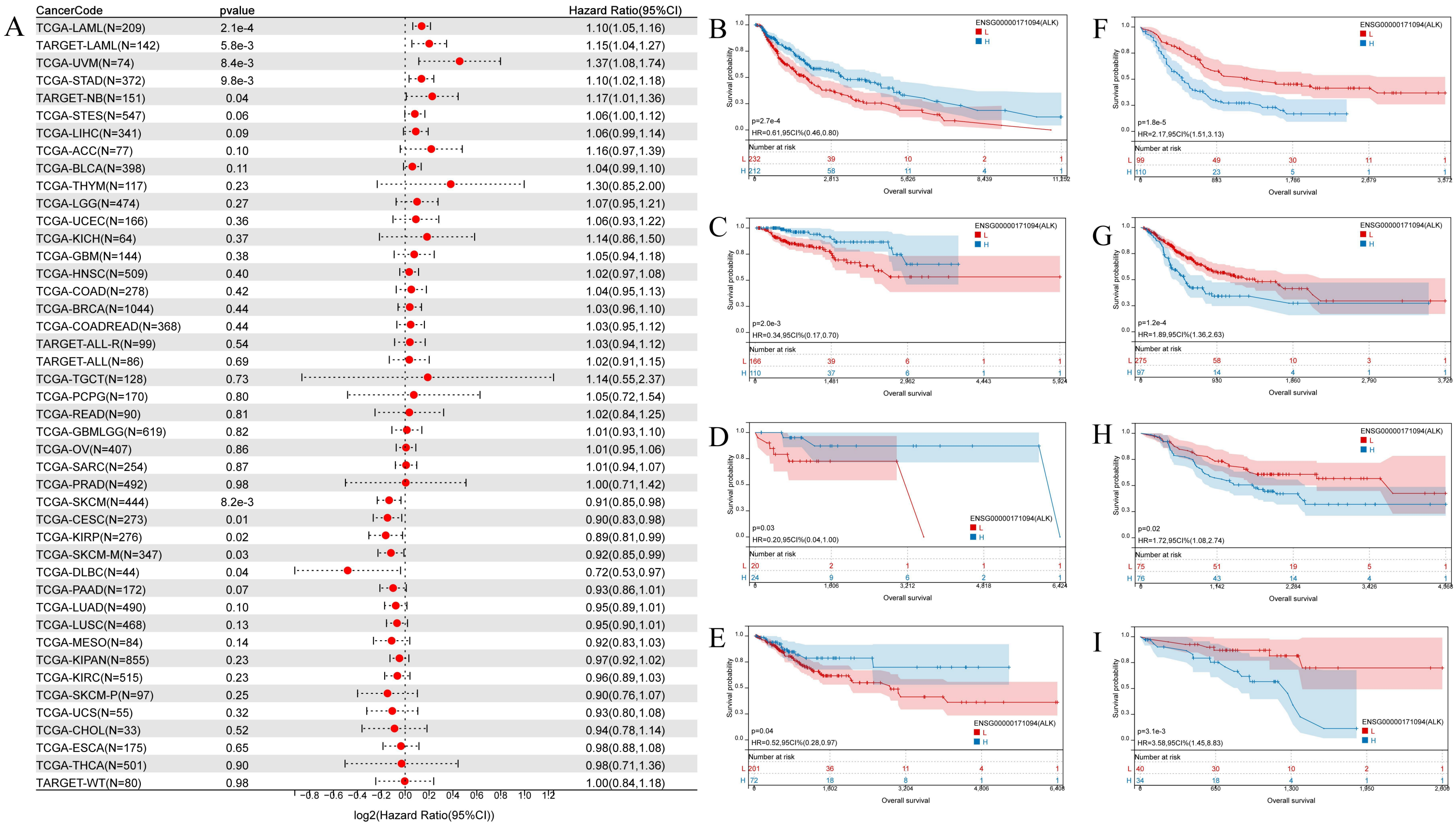

Attached figure 2: Prognostic analysis of ALK expression in pan-cancer.
A, Forest plot of prognostic analysis of ALK expression in pan-cancer;
B, Kaplan-Meier survival analysis of ALK high and low expression groups in TCGA-SKCM;
C, Kaplan-Meier survival analysis of ALK high and Low expression groups in TCGA-KIRP;
D, Kaplan-Meier survival analysis of ALK high and low expression groups in TCGA-DLBC;
E, Kaplan-Meier survival analysis of ALK high and low expression groups in TCGA-CESC;
F, Kaplan-Meier survival analysis of ALK high and low expression groups in TCGA-LAML;
G, Kaplan-Meier survival analysis of high and low expression groups in TCGA-STAD;
H, Kaplan-Meier survival analysis of ALK high and low expression groups in TCGA-NB;
I, Kaplan-Meier survival analysis of ALK high and low expression groups in TCGA-UVM.

List of abbreviations

Full name	Abbreviations
small cell lung cancer	SCLC
non-small cell lung cancer	NSCLC
Anaplastic lymphoma kinase	ALK
Anaplastic lymphoma kinase - tyrosine kinase inhibitors	ALK-TKIs
thyroid transcription factor-1	TTF-1
Neural cell adhesion molecule 1	CD56
immunohistochemistry	IHC
Response Evaluation Criteria in Solid Tumors	RECIST
Overall survival	OS
Cell Condition Solution-1	CC-1
interquartile range	IQR
proliferation cell nuclear antigen	Ki-67
Cytokeratin7	CK7
Chromogranin A	CgA
Synaptophysin	Syn
computed tomography	CT
fluorescence in situ hybridization	FISH
next-generation sequencing	NGS
Deoxyribonucleic acid	DNA
Partial Response	PR
Progression-Free Survival	PFS
Echinoderm microtubule-associated protein-like 4	EML4
Polymerase Chain Reaction	PCR
Epidermal Growth Factor Receptor	EGFR
Adrenocortical carcinoma	TCGA-ACC
Bladder Urothelial Carcinoma	TCGA-BLCA
Breast invasive carcinoma	TCGA-BRCA
Cervical squamous cell carcinoma and endocervical adenocarcinoma	TCGA-CESC
Cholangiocarcinoma	TCGA-CHOL
Colon adenocarcinoma	TCGA-COAD
Colon adenocarcinoma/Rectum adenocarcinoma Esophageal carcinoma	TCGA-COADREAD
Lymphoid Neoplasm Diffuse Large B-cell Lymphoma	TCGA-DLBC
Esophageal carcinoma	TCGA-ESCA
FFPE Pilot Phase II	TCGA-FPPP
Glioblastoma multiforme	TCGA-GBM
Glioma	TCGA-GBMLGG
Head and Neck squamous cell carcinoma	TCGA-HNSC
Kidney Chromophobe	TCGA-KICH
Pan-kidney cohort (KICH+KIRC+KIRP)	TCGA-KIPAN
Kidney renal clear cell carcinoma	TCGA-KIRC
Kidney renal papillary cell carcinoma	TCGA-KIRP

Acute Myeloid Leukemia	TCGA-LAML
Brain Lower Grade Glioma	TCGA-LGG
Liver hepatocellular carcinoma	TCGA-LIHC
Lung adenocarcinoma	TCGA-LUAD
Lung squamous cell carcinoma	TCGA-LUSC
Mesothelioma	TCGA-MESO
Ovarian serous cystadenocarcinoma	TCGA-OV
Pancreatic adenocarcinoma	TCGA-PAAD
Pheochromocytoma and Paraganglioma	TCGA-PCPG
Prostate adenocarcinoma	TCGA-PRAD
Rectum adenocarcinoma	TCGA-READ
Sarcoma	TCGA-SARC
Stomach adenocarcinoma	TCGA-STAD
Skin Cutaneous Melanoma	TCGA-SKCM
Stomach and Esophageal carcinoma	TCGA-STES
Testicular Germ Cell Tumors	TCGA-TGCT
Thyroid carcinoma	TCGA-THCA
Thymoma	TCGA-THYM
Uterine Corpus Endometrial Carcinoma	TCGA-UCEC
Uterine Carcinosarcoma	TCGA-UCS
Uveal Melanoma	TCGA-UVM
Osteosarcoma	TARGET-OS
Acute Lymphoblastic Leukemia	TARGET-ALL
Neuroblastoma	TARGET-NB
High-Risk Wilms Tumor	TARGET-WT

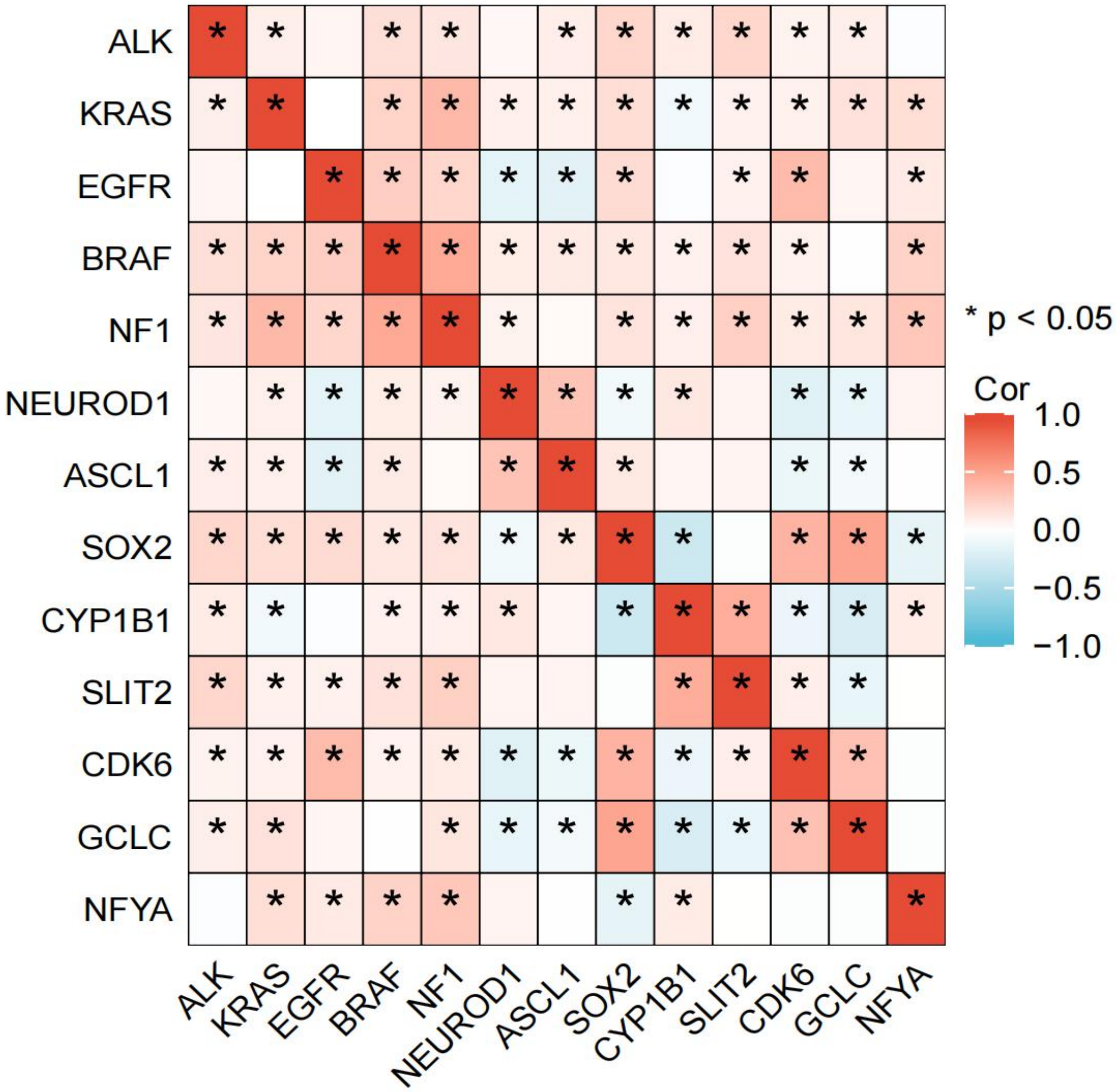

Attached figure 3: Correlation heat map of ALK, EGFR, KRAS, BRAF, NF1, NEUROD1, ASCL1, SOX2, CYP1B1, SLIT2, CDK6, GCLC, and NFYA gene expression. ALK, Anaplastic lymphoma kinase; EGFR, Epidermal growth factor receptor; KARS, Kirsten rat sarcoma viral oncogene homolog; BRAF, B-Raf proto-oncogene, serine/threonine kinase; NF1, Neurofibromatosis type 1; NEUROD1, Neurogenic Differentiation 1; ASCL1: Achaete-scute family bhlh transcription factor 1; SOX2: Sry-box transcription factor 2; CYP1B1: Cytochrome P450 1B1; SLIT2: Slit guidance ligand 2; CDK6: Cyclin-dependent kinase 6; GCLC: Glutamate-cysteine ligase catalytic subunit; NFYA: Nuclear transcription factor Y subunit alpha.
